# Structural, electronic, and optical properties of cubic formamidinium lead iodide perovskite: a first-principles investigation†

**DOI:** 10.1039/d0ra06028c

**Published:** 2020-09-01

**Authors:** Sanjun Wang, Wen-bo Xiao, Fei Wang

**Affiliations:** College of Artificial Intelligence, Henan Finance University Zhengzhou 450046 China; Key Laboratory of Nondestructive Testing Ministry of Education, Nanchang Hangkong University Nanchang 330063 China; International Laboratory for Quantum Functional Materials of Henan, School of Physics and Microelectronics, Zhengzhou University Zhengzhou 450001 China wfei@zzu.edu.cn

## Abstract

Hybrid organic–inorganic perovskites have been one of the most active areas of research into photovoltaic materials. Despite the extremely fast progress in this field, the electronic properties of formamidinium lead iodide perovskite (FAPbI_3_) that are key to its photovoltaic performance are relatively poorly understood when compared to those of methylammonium lead iodide (MAPbI_3_). In this study, first-principles total energy calculations based on density functional theory were used to investigate the favored orientation of FA. Different theoretical methods, with or without incorporation of spin-orbit coupling (SOC) effects, were used to study the structure, electronic properties, and charge-carrier effective mass. Also the SOC-induced Rashba *k*-dependent band splitting, density of states and optical properties are presented and discussed. These results are useful for understanding organic–inorganic lead trihalide perovskites and can inform the search for new materials and design rules.

## Abstract 2

Different theoretical methods, including SOC effects, were used to study the detailed structure, electronic properties, charge-carrier mobility, and SOC-induced Rashba *k*-dependent band splitting in FAPbI_3_.

## Graphical abstract

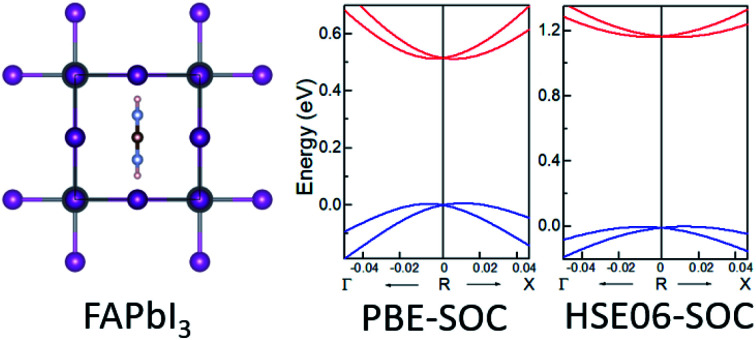

## 1. Introduction

Hybrid organic–inorganic perovskites have found prominence as a material for the active photovoltaic layer in optoelectronic devices due to their high and balanced charge-carrier mobilities, suitable band gaps, and high absorption cross sections.^1–3^ The most studied organic–inorganic lead trihalide perovskites have the general composition APbX_3_, where A = methylammonium (MA), formamidinium (FA), or Cs, and X = I, Br, or Cl. Rapid progress in perovskite photovoltaic devices has seen certified efficiencies rise to above 25.2%.^4^ Although MAPbI_3_ is the most studied hybrid metal-organic perovskite for photovoltaic applications, better performance in terms of photovoltaic efficiency is found in FAPbI_3_ or mixed FA and MA hybrid perovskites.^5–7^ This is because the increase in the effective cation radius caused by switching from the MA to the FA cation decreases the optical band gap, extending absorption into the near-infrared. Also FAPbI_3_ gives better stability than MAPbI_3_ at high temperatures.^8–10^ However, its major flaw is that the black cubic α phase, which is responsible for the high photovoltaic efficiency, is metastable toward heat and moisture at normal operating conditions.^11–13^ The stable phase, which is produced when FAPbI_3_ is obtained by standard chemical methods, is the yellow hexagonal δ phase. Recently, however, Cordero *et al.* found that α-FAPbI_3_ can be perfectly stable for at least 100 days unless extrinsic factors induce its degradation.^8^

There have been extensive theoretical works studying the structure and electronic, optical, and defect properties of MAPbI_3_, and these have greatly deepened the understanding of MAPbI_3_ and accelerated research into its application to devices.^14–19^ Although there have been some of theoretical works studying FAPbI_3_,^20–24^ a systematic and comprehensive study is still absent. Pan *et al.* investigate the geometric and electronic structures of hybrid organic–inorganic perovskites FAPbX_3_ (X = Cl, Br, I).^20^ Quarti *et al.* studied the flexibility structural and dynamics electronic properties of MAPbI_3_ and FAPbI_3_ using *ab initio* molecular dynamics simulations.^21^ Kanno *et al.* theoretically studied the rotational potential energy surface of FAPbI_3_.^22^ Liu and Yam studied its intrinsic defects.^23^ Guo *et al.* studied the effects of Rb incorporation and water degradation of the FAPbI_3_ surface.^24^

In this work, by using the first-principles total energy calculation method, we systematically investigated the structure, electronic properties, charge effective mass, *k*-dependent band splitting and optical properties of cubic α-FAPbI_3_. Different calculation methods were evaluated, specifically, standard density functional theory (DFT), screened hybrid DFT, and the *GW* approach, both with and without incorporation of spin-orbit coupling (SOC) effects. The ideal method would be a *GW* approach incorporating SOC, but this is highly computationally expensive. Among the other approaches, the DFT method using the Perdew–Burke–Ernzerhof functional (DFT-PBE) gives a more accurate band-gap energy, using the PBE functional including van der Waals interactions (PBE-vdW) gives a more accurate lattice constant, and the screened hybrid functional of Heyd, Scuseria, and Ernzerhof (HSE06) gives more reliable contributions from SOC effects. The transport properties of the charge effective mass and the optical properties are also given and discussed.

## 2. Computational details

The first-principles total energy calculation was performed using the Vienna *Ab initio* Simulation Package (VASP)^25,26^ with the standard frozen-core projector augmented-wave method and the exchange–correlation functional of generalized gradient approximation in the PBE format.^27^ A cut-off energy of 400 eV was chosen to achieve the desired accuracy. During the optimization of the geometric structure, the total energy convergence criterion was chosen as 10^−5^ eV, and the force on each atom was converged to an accuracy of 0.01 eV Å^−1^. The zero-damping DFT-D3 method of Grimme^28^ was amended to correct for the vdW interaction in the DFT-vdW calculations. Since relativistic effects has large effect on the Pb atom, this effect was considered using SOC calculations. The HSE06 functional^29^ with 25% of the Hartree–Fock exchange was used in the HSE06 calculations. A reciprocal-space sampling with Γ centers in an 8 × 8 × 8 Monkhorst–Pack *k*-point mesh^30^ was set in the Brillouin zone for the standard DFT calculations. For the HSE06 calculations, a shifted 3 × 3 × 3 *k*-point mesh was set in the Brillouin zone. The VASPKIT toolkit was used to obtain the lattice constant, the bulk modulus in the Birch–Murnaghan equation of state, and the charge effective mass.^31^

## 3. Results and discussion

### 3.1 Structural properties

**Fig. 1:**
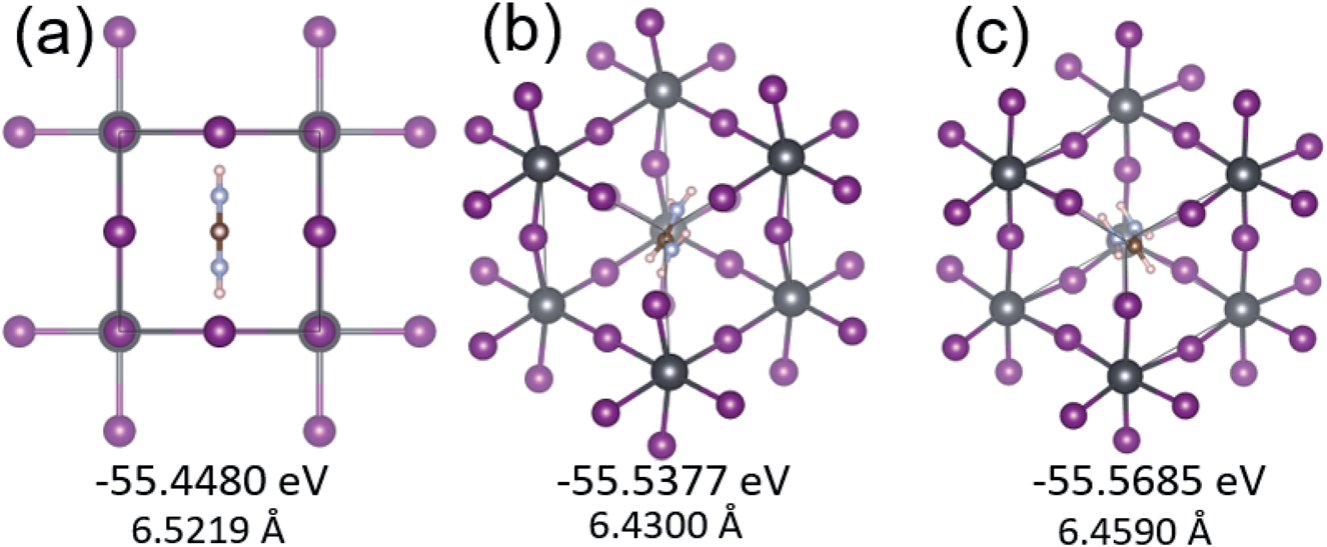
The optimized structures of FA aligned with the origin in the (a) 〈100〉, (b) 〈110〉, and (c) 〈111〉 directions in FAPbI_3_. The labels below each panel indicate the optimized total energy and the lattice constant of the unit cell.

At 300 K, FAPbI_3_ has a cubic perovskite ABX_3_ structure with space group *Pm*3̄*m*. Although FA molecules are less polar than MA molecules, the polarization of FA still has great impact on the structure and the optoelectronic properties of FAPbI_3_, such as the total energy, the crystal lattice, phase transitions, and the band gap of the system. To clarify the favored orientation of the FA cation in FAPbI_3_, we performed first-principles total-energy calculations on various FA orientations. Three possible local minima were set, with the FA molecule aligned along the 〈100〉, 〈110〉, and 〈111〉 directions. In contrast to the single C–N bond in MA, the direction of polarization in MA is unique, there are two C–N bonds in the FA molecule and the N–C–N group forms a 126° angle. We therefore used the connection directions of the two nitrogen atoms to align the FA molecule inside the unit cell. Aside from those forming the fixed cell shape, all atoms within the cell included the volume were fully relaxed to obtain the energy minimum. Here, the 〈100〉 orientation structure was obtained from the well-accepted structure of Weller *et al.*^12^ The 〈110〉 and 〈111〉 structures were obtained by rotating the FA molecule into each respective orientation. The fully optimized structures are shown in Fig. 1, and the total energy of the unit cell and its lattice constant are shown below each structure. It can be seen from Fig. 1(b) that in the 〈110〉 structure, the FA molecule relaxed to the 〈111〉 orientation with slight divergence. The total energy of the optimized systems was found to be lower than in the 〈100〉 structure, by 0.0897 eV and 0.1205 eV. Furthermore, the band gaps, at 1.6316 and 1.6842 eV, were larger than the 1.5434 eV found in the 〈100〉 structure. So there are two local minimum total energy structure configuration in FAPbI_3_, that are FA aligned with the 〈100〉 orientation or 〈111〉 orientation, as shown in Fig. 1.

The ground state of FAPbI_3_ with a 〈111〉 orientation was the most stable, which is consistent with the results of calculations with the MA cation in the ground state of MAPbI_3_, due to the organic molecule being oriented in the 〈111〉 direction, where it has maximum freedom.^32,33^ The most stable 〈111〉 orientation structure was different from the 〈100〉 orientation structure obtained by Weller *et al.*^12^ This can be explained by Weller *et al.*'s structure being obtained at 298 K, whereas our DFT-calculated 〈111〉 orientation was the ground state at 0 K. This divergence in the 〈111〉 orientation at low temperature may induce a locally disordered low-temperature γ phase,^13^ while the regular 〈100〉 orientation of the FA molecule results in a locally ordered high-temperature α phase. This rotational dynamics of FA molecular have revealed by the first-principles molecular dynamics simulation by Carignano *et al.*,^34^ which show that at room temperature, the orientation of the sum vector is essentially (100) and should form a rotational glass at lower temperatures. These thermal effects of FA molecular are similar with MA molecular in MAPbI_3_.^35,36^ Since only the cubic α phase at room temperature is of interest for use in solar cells, we examine only the 〈100〉 orientation cubic α phase in experiment by Weller *et al.*^12^ in the remainder of this paper.

### 3.2 Electronic properties

Geometric optimization of the atomic positions (and cell parameters) was performed using three different methods, that is, DFT-PBE, DFT-vdW, and HSE06. The lattice parameters, bulk modulus, and band gap, with and without SOC, of the cubic FAPbI_3_ structure obtained from our calculations are listed in Table 1. Comparing the calculations from the three different models, DFT-PBE provides the most accurate band gap, 1.5434 eV compared with the experimental results of 1.53 eV to 1.45 eV. However, this accuracy is mainly due to the strong relativistic effects of the Pb atom offsetting the underestimation error in the band gap obtained using typical DFT calculations. It can be seen from Table 1 that after including SOC, the DFT-PBE method gives a band gap of only 0.5157 eV, and the DFT-vdW method gives a similarly low band gap of 0.4169 eV. The HSE06-SOC method produces a value of 1.1732 eV, which is still significantly lower than the experimental value.

**Table 1:** Calculated lattice constant *a*, bulk modulus *B*_0_, and band gap *E*_g_ with and without SOC, of cubic FAPbI_3_. Corresponding results from the theoretical and experimental literature are also presented for comparison

	Lattice constant *a* (Å)	Bulk modulus *B*_0_ (GPa)	Band gap *E*_g_ (eV)	*E*_g_ with SOC (eV)
DFT-PBE	6.5219	11.5287	1.5434	0.5157
DFT-vdW	6.4064	16.1087	1.3952	0.4169
HSE06	6.5193	11.3210	1.9937	1.1732
Theoretical	6.3992,^20^ 6.42,^24^	—	1.368,^19^ 1.40,^20^ 1.75,^21^ 1.45,^24^ 1.43,^37^ 1.43,^38^	0.224,^19^ 0.66,^21^ 0.82,^24^ 1.47,^38^
Experimental	6.3503,^11^ 6.3620,^37^ 6.3558,^39^	—	1.53,^11^ 1.489,^9^ 1.45,^39^	—

To obtain a more accurate value for the band gap, we used the *GW* approach. Due to the high computational demands of the technique, we did not include SOC in these *GW* calculations. Using the DFT-vdW lattice constant, we obtained a band gap of 2.6041 eV from the *GW* approach. If the decrease of ∼1.0 eV resulting from the SOC effect in the DFT calculations were repeated with the *GW* calculations, that is, the reduction from 1.5434 to 0.5157 eV in DFT-PBE and from 1.3952 to 0.4169 eV in DFT-vdW, the *GW* method including SOC would give a band gap of 1.6041 eV, which is close to the experimental value of 1.45 to 1.53 eV. If computing resources did not need to be considered, the *GW* approach incorporating SOC would give the best results when compared to the experimental value. Otherwise, the DFT-PBE method results in the most accurate band-gap energy, the PBE-vdW method provides the most accurate lattice constant, and the HSE06 method gives reliable calculations of the contributions from SOC effects.

**Fig. 2:**
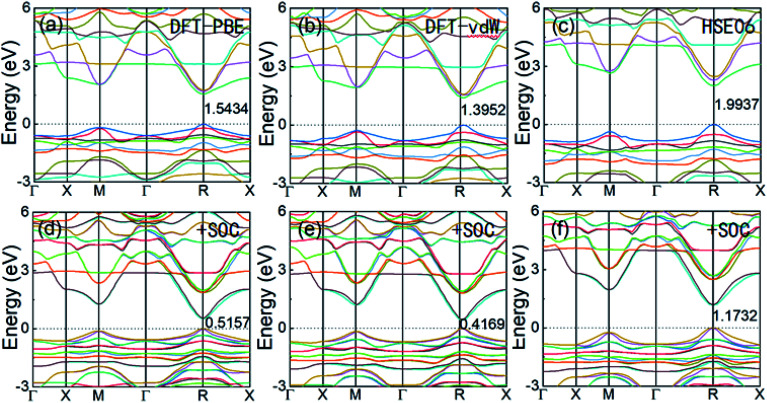
The electronic band structures of perovskite cubic FAPbI_3_ calculated from DFT-PBE, DFT-vdW, and HSE06, without SOC (a–c) and with SOC (d–f).

Cubic α-FAPbI_3_ perovskite is a direct-band-gap semiconductor with its conduction band minimum (CBM) and its valence band maximum (VBM) at the same R point (0.5, 0.5, 0.5). Fig. 2 shows the three different calculated band structures with and without the inclusion of SOC effects. It can be seen that the inclusion of SOC effects greatly decreases the band gap of FAPbI_3_. The band gaps calculated from DFT-PBE and DFT-vdW are decreased by about 1.0 eV after inclusion of the SOC effects. The HSE06 calculation including SOC effects has a band gap that is about 0.82 eV lower, decreasing from 1.9937 eV to 1.1732 eV. The good consistency of the band-gap energy with experiments in the DFT calculations is caused by the strong relativistic effect of the Pb atoms offsetting the underestimation of the band gap by typical DFT calculations. This accurate calculation of band-gap energy is also seen in the typical Pb-containing perovskite, MAPbI_3_.^15,32^ Considering that the three sets of calculations with and without the inclusion of SOC effects result in nearly the same band-edge orbital characters and band-gap positions in *k*-space, as well as the same trend in band-gap change, it can be concluded that DFT-PBE is able to provide an accurate qualitative picture of the evolution of the electronic structure in FAPbI_3_ in common cases.

**Fig. 3:**
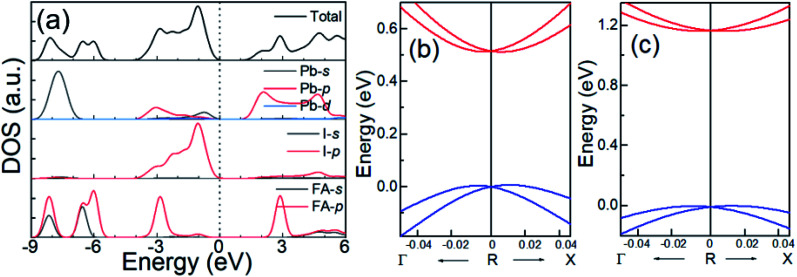
(a) The total and projected DOS of FAPbI_3_ for Pb, I, and FA from DFT-PBE calculations, and enlarged views of the band dispersion around the R point for (b) DFT-PBE with SOC and (c) HSE06 with SOC.

Fig. 3(a) shows the total and projected density of states (DOS) of cubic FAPbI_3_ calculated using the DFT-PBE method. Based on these calculations, in FAPbI_3_, FA and Pb donate one and two electrons, respectively, to the three I ions, forming a band gap between the unoccupied Pb 6p orbital in the conduction band (CB) and the occupied Pb 6s and I 5p orbitals in the valence band (VB), which is consistent with the MAPbI_3_ results.^14,32^ There are, however, differences in orbital character that result from the differences between the FA and MA molecules. The FA shows DOS peaks near −2.9 and 3.0 eV by the 2p orbitals of the C and N atoms, while the MA shows orbitals near and below −5 eV, under the Fermi level, and no orbitals in the CB.^1,14^

Because SOC has a large effect on the band-gap energy, it is an interesting property that produces *k*-dependent band splitting in Pb-containing perovskites. Enlarged views of the band dispersion around the R point as calculated by DFT-PBE and HSE06 with SOC are shown in Fig. 3(b) and (c), respectively. It can be seen that the VB and CB both exhibit band splitting, and this seems to be the same in the CB and the VB. This is in contrast to previous results obtained for MAPbI_3_ because previous studies used the lower-symmetry tetragonal or pseudocubic phases.^16^ Here, we used the cubic *Pm*3̄*m* phase. Our results show that Pb SOC effects induce a large decease in the band gap and cause band splitting in the band edge of FAPbI_3_. These large SOC effects and Rashba band splitting may induce a long carrier life in FAPbI_3_.^40^

### 3.3 Transport properties

The remarkable properties of FAPbI_3_ perovskite in relation to solar cells are partially the result of its excellent charge–transport properties. Using the parabolic approximation, we calculated the effective mass (m*) of carriers around the CBM and the VBM by fitting the dispersion relation, m* = ℏ^2^/[∂^2^*E*(*k*)/∂k^2^], where *E*(*k*) is the band-edge eigenvalue and *k* is the wavevector. The effective hole and electron masses 
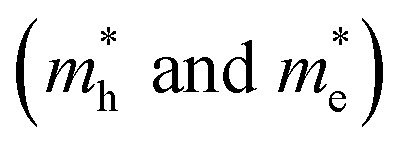
 along three high-symmetry directions in cubic FAPbI_3_, calculated using the DFT-PBE and HSE06 methods with and without the inclusion of SOC effects, are shown in Table 2. If the spin-orbit coupling effects are included, the effective mass decreases markedly. The lowest effective mass reaches 0.15 *m*_0_ for both holes and electrons. We get an average effective hole and electron masses 0.213 *m*_0_ and 0.184 *m*_0_ close with Muhammad *et al.*^38^*G*_0_*W*_0_ + SOC results 0.273 *m*_0_ and 0.218 *m*_0_. These results show that the SOC effects have a great impact on not only the band-gap energy but also band-edge dispersion and transport properties. This is consistent with the results for MAPbI_3_.^16,32^ Furthermore, the smaller effective mass in FAPbI_3_ as compared to MAPbI_3_ will result in better optoelectronic properties. This improvement in charge–transport properties and the smaller band gap in FAPbI_3_ than MAPbI_3_ results in better optoelectronic properties in cubic FAPbI_3_.

**Table 2:** The effective hole and electron masses 
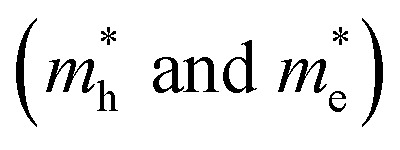
 along three high-symmetry directions in cubic FAPbI_3_ as calculated by DFT-PBE and HSE06 with and without the inclusion of SOC effects. The unit is *m*_0_

	DFT-PBE		DFT-PBE + SOC		HSE06		HSE06 + SOC		*G*_0_ *W*_0_ + SOC^38^	
	*m*_h_	*m*_e_	*m*_h_	*m*_e_	*m*_h_	*m*_e_	*m*_h_	*m*_e_	*m*_h_	*m*_e_
R-Γ	0.200	0.255	0.147	0.109	0.214	0.265	0.241	0.144	—	—
R-X	0.239	0.188	0.186	0.115	0.270	0.195	0.190	0.149	—	—
R-M	0.192	0.843	0.143	0.116	0.251	0.848	0.209	0.260	—	—
Average	0.210	0.428	0.159	0.113	0.245	0.436	0.213	0.184	0.273	0.218

### 3.4 Optical properties

**Fig. 4:**
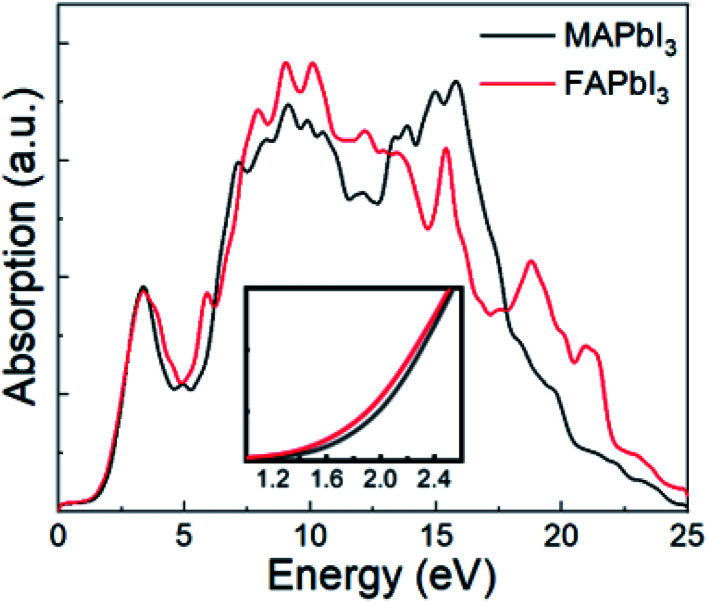
Absorption spectra for FAPbI_3_ and MAPbI_3_. Inset shows an enlarged plot of the absorption near the band gap.

As FAPbI_3_ is an important optoelectronic material for solar cells, it is useful to study its absorption spectrum. For comparison, Fig. 4 shows absorption spectra for both FAPbI_3_ and MAPbI_3_ as calculated by DFT-PBE, which can reflect the absorption ratio already. It can be seen that FAPbI_3_ has almost the same high absorption spectrum as MAPbI_3_. From the inset, it can also be seen that FAPbI_3_ has a lower band gap than MAPbI_3_. The high level of absorption seen in these spectra is what leads to the high efficiency seen in mixed FA and MA solar cells.

## 4. Conclusions

This study systematically examined the structure, electronic properties, charge effective mass, and optical properties of cubic α-FAPbI_3_. The calculations of total energy showed that the most stable FA structure is aligned in the 〈111〉 direction at 0 K, though this reorients to a 〈100〉 ordered-symmetry structure at high temperatures. Different theoretical methods, specifically standard DFT-PBE and PBE-vdW, HSE06, and *GW*, with and without the incorporation of SOC effects, were evaluated in this work. The ideal method would be a *GW* approach incorporating SOC. Among the other methods, DFT-PBE results in a more accurate band-gap energy, DFT-vdW gives a more accurate lattice constant, and HSE06 gives more reliable calculations of the contributions from SOC effects. The SOC effects have a great impact on the band-gap energy and Rashba band splitting, which may induce a long carrier life in FAPbI_3_. The excellent and balanced charge-carrier motilities and optical-absorption properties explain the high photovoltaic efficiency in cubic α-FAPbI_3_. These results are useful for understanding organic–inorganic lead trihalide perovskites and can inform the search for new materials and design rules.

## Conflicts of interest

There are no conflicts to declare.

## Supplementary Material

RA-010-D0RA06028C-s001
