# Supplementary material for: Structural, electronic, and optical properties of cubic formamidinium lead iodide perovskite: a first-principles investigation
Source: RSC Adv. 2020 Sep 1;10(54):32364–9. doi: 10.1039/d0ra06028c (PMC9056641; doi:10.1039/d0ra06028c)
Supplement: RA-010-D0RA06028C-s001 [file RA-010-D0RA06028C-s001.pdf]

Electronic Supplementary Material (ESI) for RSC Advances.
This journal is © The Royal Society of Chemistry 2020

Supporting information:

Structural, electronic, and optical properties of cubic formamidinium lead iodide perovskite: A first-principles investigation

Sanjun Wang[a], Wen-bo Xiao[b], Fei Wang[c,*]

[a] *College of Artificial Intelligence, Henan Finance University, Zhengzhou 450046, China;*

[b] *Key Laboratory of Nondestructive testing, Ministry of Education, Nanchang Hangkong University, Nanchang 330063, China;*

[c] *International Laboratory for Quantum Functional Materials of Henan, School of Physics and Microelectronics, Zhengzhou University, Zhengzhou 450001, China.*

The POSCAR file for $FAPbI_3$ PBE [111].

```
FAPbI3 PBE [111]
   1.00000000000000
     6.4589972925718193    0.0000000000000000    0.0000000000000000
     0.0000000000000000    6.4589972925718193    0.0000000000000000
     0.0000000000000000    0.0000000000000000    6.4589972925718193
   C    N    H    Pb    I
     1    2    5    1    3
Direct
  0.4446100039999976  0.4784399710000002  0.5249500150000017
  0.4421560795451001  0.6094450370013220  0.3706999280163501
  0.5863716296305626  0.4718834382313304  0.6686733616323792
  0.3189267628053969  0.3661898113936886  0.5342151560554598
  0.3247655868173496  0.6012420605569488  0.2649740724251757
  0.5524830491180950  0.7206484476027484  0.3514892894933936
  0.7094075923344363  0.5702758137779185  0.6715301513277225
  0.5768870461671369  0.3618426087835545  0.7817959997122372
  0.0000000000000000  0.0000000000000000  0.0000000000000000
  0.4955008036196986  0.0340738133587379  0.0084927291793448
  0.0104307130704626  0.5068825151667298  0.0309936222465907
  0.9559045289805326  0.9716826665041225  0.5159527464927507
```

* Email:wfei@zzu.edu.cn
